# Henry William Collins

**Published:** 1906-03

**Authors:** 


### HENRY WILLIAM COLLINS, M.R.C.S., L.S.A.

Mr. Collins, of Wrington, was the eldest son of the late Rev. E. Collins, of Cowslip Green, Wrington, and was born at Geelong, Victoria, in the year 1847. He was brought over to England at an early age, was educated at the Godolphin Grammar School, Hammersmith, was then apprenticed to Dr. Horace Swete, who was at that time in practice in Wrington, and later on went through the curriculum at Guy's Hospital, where he devoted himself to his medical studies, also to football, rowing and swimming. He took his L.S.A. in 1870, and his M.R.C.S. in 1871. He then returned to Wrington, and bought the practice to which he had been apprenticed. Later on he joined with his *confrère*, the late Dr. Chadwick, and continued in practice until October, 1902, when he retired owing to failing health. He did not enjoy his retirement long, as he was soon after stricken with the disease which recently proved fatal in his 59th year.

Among Freemasons his loss will be as deeply felt as it will be among his medical brethren. He was a distinguished member of that body, and held high rank in the Province of Somerset.

In character he was a man who possessed to a peculiar degree the power of making himself universally loved, and it was that power, combined with his medical skill, which contributed so largely to his undoubted success.

He was always keen on his medical work, was an excellent practitioner, and a frequent attendant at medical meetings in Bristol.

